# Age over 65 years and high levels of C-reactive protein are associated with the risk of preoperative deep vein thrombosis following closed distal femur fractures: a prospective cohort study

**DOI:** 10.1186/s13018-020-02089-4

**Published:** 2020-11-25

**Authors:** Junzhe Zhang, Kuo Zhao, Junyong Li, Hongyu Meng, Yanbin Zhu, Yingze Zhang

**Affiliations:** 1 Department of Orthopaedic Surgery, the Third Hospital of Hebei Medical University, No. 139 Ziqiang Road, Shijiazhuang, 050051 Hebei Province People’s Republic of China; 2 Key Laboratory of Biomechanics of Hebei Province, Orthopaedic Research Institution of Hebei Province, Shijiazhuang, Hebei Province People’s Republic of China; 3 Department of Orthopedic Surgery, the Second Hospital of Shijiazhuang City, Shijiazhuang, Hebei Province People’s Republic of China

**Keywords:** Predictor, Old age, C-reactive protein, Deep vein thrombosis, Distal femoral fracture

## Abstract

**Background:**

In this study, we investigated the epidemiological characteristics and predictors of preoperative new-onset deep vein thrombosis (DVT) in adult patients with closed distal femur fractures (DFFs).

**Methods:**

The study was designed as a prospective cohort trial at the Third Hospital of Hebei Medical University. From October 2018 to June 2020, a total of 160 patients with closed DFFs were enrolled to assess the location and prognosis of preoperative DVT. The patients were followed up for 2 months. Duplex ultrasonography (DUS) was used to diagnose patients with DVT. The patients were divided into two groups (DVT group and non-DVT group). The DVT was then classified into proximal, distal, and mixed thromboses. The Mann-Whitney *U* test or *t* test, receiver operating characteristic (ROC) analyses, univariate Chi-square analyses, and multiple logistic regression analyses were used to analyze the adjusted predictors of DVT.

**Results:**

The overall incidence of preoperative DVTs was 52.5% (*n* = 84), which was diagnosed at a mean period of 3.1 days after injury. Among patients diagnosed with DVTs, 50.0% (*n* = 42) had distal thrombosis while 47.6% (*n* = 40) had mixed thrombosis. The calf muscle veins were the most common sites of DVTs (90.5%, *n* = 76). Of note, 45.2% (*n* = 38) of diagnosed DVTs were completely recanalized at a mean period of 12.0 days after the initial (first) diagnosis. Multivariate analysis revealed that age of ≥ 65 years of age (odds ratio [OR], 4.390; 95% confidence interval [CI] 1.727–11.155; *p* = 0.002), C-reactive protein (CRP) levels exceeding 11 mg/L (OR 4.158; 95% CI 1.808–11.289; *p* = 0.001), platelet (PLT) levels over 217 × 109/L (OR, 2.55; 95% CI 1.07–6.07; *p* = 0.035), D-dimer levels over 1.0 mg/L (OR 3.496; 95% CI 1.483–8.237; *p* = 0.004), and an American Society of Anesthesiologists (ASA) score of III-V (OR 2.753; 95% CI 1.216–6.729; *p* = 0.026) were the independent risk factors of preoperative DVT.

**Conclusions:**

High levels of CRP, PLT, D-dimer, ASA, and ≥ 65 years of age increase the risk of preoperative DVTs in adult patients with closed DFFs. Thus, the prediction of preoperative DVTs can significantly be improved by identifying older patients over the age of 65, and establishing the biochemical cut-off values of CRP, PLT, ASA, and D-dimer.

**Trial registration:**

No. 2018-026-1, 24 October 2018, prospectively registered.

This trial was registered prospectively on 24 October 2018 before the first participant was enrolled. This study protocol conformed to the Declaration of Helsinki and approved by the Institutional Review Board. The ethics committee approved the study on the factors of prognosis for patients with fractures. Data used in this study were obtained from the patients who underwent orthopedic surgery between October 2018 and June 2020.

## Background

Distal femur fractures (DFFs) are relatively uncommon but fatal. DFFs comprise approximately 8.7% and 0.8% of all femoral fractures and body fractures in Chinese adults, respectively [1]. Majority of DFFs cases in older patients is caused by low-energy injures such as fall-related traumas. Its annual mortality rate is 13.4% [2]. Geriatric DFFs are the second most prevalent fragility fractures after hip fractures [3, 4], and are accompanied by several complications [5, 6]. DFFs affects the articular surface and vascular or nerve injuries. Hence, DFF inevitably leads to knee dysfunction, traumatic arthritis, bone nonunion, venous thromboembolism (VTE), and other perioperative complications. Deep venous thrombosis (DVT) is difficult to treat given that is it associated with immune and inflammatory cells activation, which keeps the intravascular system in a hypercoagulability state, which leaves patients at a higher risk of developing DVT. The 1-month mortality of DVT was 4.6%, much higher as compared to that of the general population [7]. The development of VTE often leads to readmission of 1.2% of fracture patients [8].

Epidemiologic characteristics of DVT following fractures affect the success of prevention and treatment strategies. It is therefore important to determine factors that predict DVT to identify patients at risk of developing DVT. Several studies have investigated the incidence, predilection sites, and factors that predict DVT after hip fracture [9, 10], joint arthroplasty [11, 12], and ankle trauma [13, 14]. However, such studies are limited in terms of study design, insufficient preoperative DVT data, and short follow-up of postoperative prognosis. In addition, the epidemiological characteristics of preoperative DVTs after DFFs are not well understood. The majority of DVT cases typically starts from the calf veins and propagate proximally [15]. Prophylactic treatment of DVT is controversial.

This prospective study was designed for two main objectives: to summarize the epidemiological features of preoperative DVTs after closed DFFs with a 2-month follow-up; and to identify preoperative DVT-related predictors and find the optimal cut-off values of continuous variables.

## Materials and methods

### Study design

**Fig. 1:**
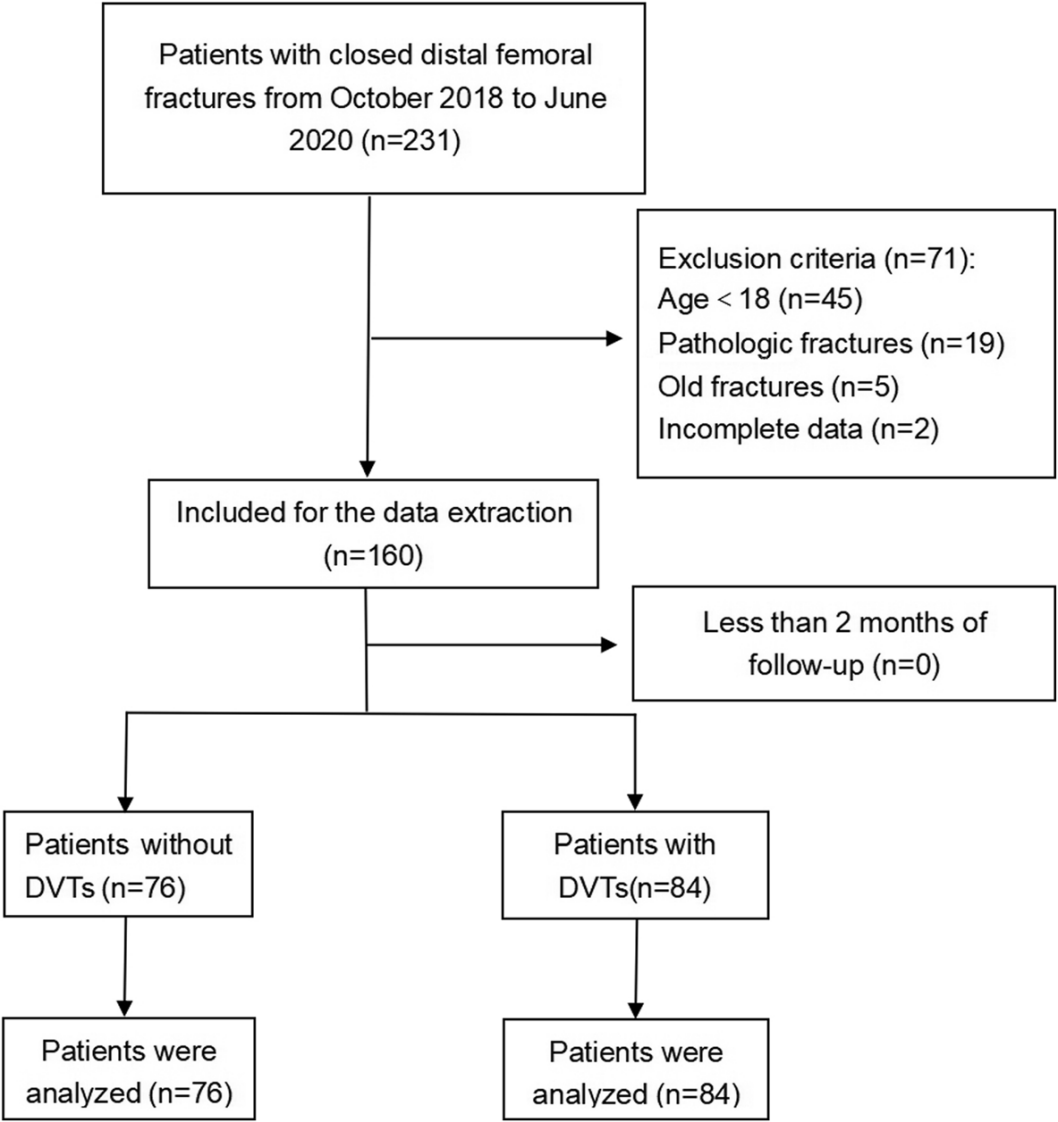
The flow chart for the selection of study participants

The present study, which was conducted from October 2018 to June 2020 at the Third Hospital of Hebei Medical University, is a prospective single-center study involving a total of 160 patients with closed DFFs. The study protocol was carried out according to the Declaration of Helsinki and approved by the Institutional Review Board (no. 2018-026-1). All participants in the study signed written informed consent before the study was conducted. The exclusion criteria were (i) < 18 years of age; (ii) old fractures (> 21 days from initial injury); (iii) open or pathological fractures; (iv) history of femur surgery and deep vein thrombosis (DVT); and (V) recent use of antithrombotic drugs (low molecular weight heparin and others), and patients with incomplete medical records. Patients with multiple closed fractures were enrolled to the study to investigate its effects on DVTs. Fig. 1 showed that 160 participants with closed DFFs were finally enrolled in the study.

### Definition and detection of DVT

Duplex ultrasonography (DUS) was used to diagnose DVT according to the Robinov group’s criteria [16]. The criteria for the diagnosis of DVT were non-compressed vein, lumen obstruction or filling defect, the lack of respiratory vibration above the knee vein segment, and inadequate flow augmentation to the calf. DUS was used to scan participants for bilateral lower-extremity DVTs after admission. Therapeutic or prophylactic thromboembolic agents were routinely administered based on the DUS result. Next, participants were reexamined by DUS every 3 days. DVTs were classified into three types: proximal (popliteal, femoral, and iliac veins), distal (calf muscle, fibular, and anterior/posterior tibial veins), and mixed DVT (both proximal and distal thrombosis).

### Data acquisition and variables of interest

Four orthopedic surgeons who underwent a similar training recorded the data mentioned below. The surgeons closely observed the patients during morning ward rounds while reviewing their clinical data. The outcomes of DVT after admission were followed for 2 months. Complex variables of interest were divided into three aspects.

Demographic variables were age (years), gender, body mass index (BMI, kg/m^2^), living place (rural, urban), cigarette consumption, alcohol consumption, diabetes mellitus, hypertension, cardiovascular disease, and previous surgeries at any body part. BMI was split into four groups based on the Chinese reference criteria: underweight, < 18.5; normal, 18.5 to 23.9; overweight, 24 to 27.9; obese, and ≥ 28 kg/m^2^.

Fracture-related variables included fracture type according to AO/OTA classification system, concurrent fracture sites (single fracture and multiple fractures), fracture side (left or right), the American Society of Anesthesiologists (ASA, I-II, and III-IV) score, and injury mechanisms. The injury mechanisms were, however, grouped into two categories: low-energy (fall from a standing height) and high-energy (traffic accidents, falling accidents from high places, human violence, and others).

Associated laboratory variables were obtained within 24 h of admission and conventionally divided into three, namely: above, below, and standard reference range. These biochemistry indices included hemoglobin (HGB), red blood cell (RBC), white blood cell (WBC), blood platelet (PLT), total serum protein (TP), albumin (ALB), alanine transaminase (ALT), aspartate transaminase (AST), C-reactive protein (CRP), prothrombin time (PT), activated partial thromboplastin time (APTT), fibrinogen (FIB), and D-dimer.

### Statistical analysis

Statistical analyses were performed using SPSS version 25.0 (IBM Corp., Armonk, NY, USA). Continuous variables were presented as median, mean ± standard deviation (SD), and range. Data normality was determined using the Shapiro-Wilk test. A Mann-Whitney *U* test or Student’s *t* test was performed to compare continuous variables between SSI and non-SSI groups according to the homogeneity of variance test and normality test. For the continuous variables with statistical significance (*p* < 0.05), receiver operating characteristic (ROC) analyses were performed to detect the optimum cut-off value, which was calculated by maximizing the sum of sensitivity and specificity in the ROC curve. Based on the cut-off value, continuous variables were converted into categorical variables before being subjected to logistic regression. The Pearson chi-square test was used to determine correlations between each categorical variable and the preoperative DVT risk. Predictors found to be significant (*p* < 0.05) in the single factor analysis were subjected to stepwise multiple logistic regression analyses (backward LR) to screen for the adjusted factors. The odds ratio (OR) and 95% confidence interval (CI) were determined to evaluate the correlation magnitude between factors and DVT risk. *p* < 0.05 was considered to be statistically significant. The Hosmer-Lemeshow test was performed to assess the fitness for the final model.

## Results

### Participant selection

Figure 1 shows the flow chart that represents the procedures used for screening the study participants. During the investigation, a total of 231 DFF patients were admitted to our institution. Among them, 45 patients were < 18 years of age; nineteen had pathological fractures (including bone or joint tumor, or soft tissue tumor); five had old fractures, while two patients had an incomplete clinical data. A total of 160 patients were finally enrolled in this study. The average age of the enrolled patients was 58.82 ± 16.01 years, while their average BMI was 26.18 ± 4.29 kg/m^2^. This cohort was made up of 59 males and 101 females, with 93 left-side and 67 right-side fractures.

### Frequency of preoperative DVTs

**Fig. 2:**
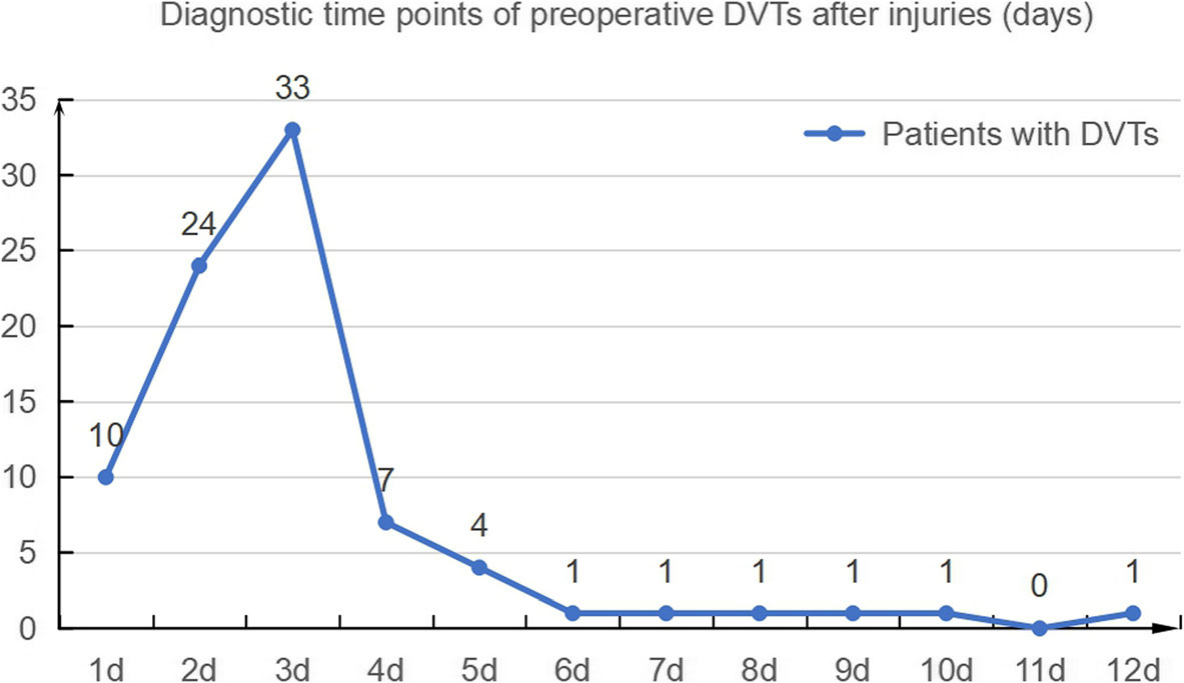
Diagnostic time points of preoperative DVTs after injuries

Figure 2 shows the diagnostic time points of the preoperative DVTs after injuries. The overall preoperative DVT incidence was 52.5% (*n* = 84), which was diagnosed at 3.06 ± 1.91 days after injury. The administration of antithrombotic agents immediately after diagnosis led to complete DVT recanalization in 45.2% (*n* = 38) of the patients at 11.89 ± 5.80 days. Table 1 shows the locations of the preoperative DVTs. Among the DVT patients, 50.0% (*n* = 42) were distal thromboses, while 47.6% (*n* = 40) were mixed thromboses. Calf muscle veins were the most common sites for DVTs (90.5%, *n* = 76).

**Table 1:** The incidence and thrombosis locations of preoperative DVTs

Location of preoperative DVTs	No. of patients (%, ***n*** = 160)
ProximalDVTs	2 (1.3%)
Distal DVTs	42 (26.3%)
Mixed DVTs	40 (25.0%)
Common femoral vein	1 (0.6%)
Deep femoral vein	2 (1.3%)
Superficial femoral vein	10 (6.3%)
Popliteal vein	37 (23.1%)
Peroneal vein	44 (27.5%)
Posterior tibial vein	49 (30.6%)
Calf muscle vein	76 (47.5%)

*DVT* deep vein thrombosis

### Continuous variables and the optimum cut-off value

Table 2 shows comparisons among the fifteen continuous variables in the DVT groups. There were significant differences in age, PLT, ALT, AST, CRP, and D-dimer between the two groups. Table 3 shows the area under the curve and the optimum cut-off values for the six continuous variables. The optimum cut-off values for age, PLT, ALT, AST, CRP, and D-dimer were 65.5 years, 217.1 × 10^9^/L, 21.5 U/L, 18.5 U/L, 11.2 mg/L, and 1.0 mg/L, respectively. Based on these cut-off values, we dichotomized the six variables for further analysis.

**Table 2:** Comparison of continuous variables in patients with and without preoperative DVTs

Variables	Patient without DVT (mean ± SD) (***n*** = 76)	Patient with DVT (mean ± SD) (***n*** = 84)	***p*** value
Age (years)	51.89 ± 14.86	65.08 ± 14.42	< 0.001^a^*
BMI (kg/m^2^)	26.05 ± 4.17	26.30 ± 4.41	0.679^b^
HGB (g/L)	119.41 ± 15.19	115.83 ± 12.03	0.112^b^
RBC (10^9^/L)	3.55 ± 0.68	3.42 ± 0.49	0.145^b^
WBC (10^9^/L)	9.39 ± 3.13	9.23 ± 3.17	0.623^b^
PLT (10^9^/L)	234.31 ± 85.36	266.80 ± 98.57	0.021^b^*
TP (g/L)	60.89 ± 6.10	59.22 ± 5.04	0.060^a^
ALB (g/L)	36.18 ± 5.18	34.67 ± 5.37	0.072^a^
ALT (U/L)	22.45 ± 13.18	33.07 ± 37.60	0.022^b^*
AST (U/L)	23.97 ± 15.95	28.51 ± 19.26	0.041^b^*
CRP (mg/L)	18.65 ± 23.22	43.31 ± 42.19	< 0.001^b^*
PT (s)	11.63 ± 0.88	11.88 ± 1.25	0.559^b^
APTT (s)	29.52 ± 3.78	29.45 ± 4.31	0.594^b^
FIB (g/L)	3.41 ± 0.78	3.47 ± 1.05	0.967^b^
D-dimer (mg/L)	1.03 ± 1.02	1.70 ± 1.65	< 0.001^b^*

*DVT* deep vein thrombosis, *BMI* body mass index, *HGB* hemoglobin, *RBC* red blood cell, *WBC* white blood cell, *PLT* platelet, *TP* total protein, *ALB* albumin, *ALT* alanine transaminase, *AST* aspartate transaminase, *CRP* C-reactive protein, *PT* prothrombin time, *APTT* activated partial thromboplastin time, *FIB* fibrinogen

*Statistical significance

^a^ Student’s *t* test

^b^ Mann-Whitney *U* test

**Table 3:** The ROC curve analysis of continuous variables with statistical significance

Variable	Cut-off value	Area under the curve (95% CI)	Sensitivity	Specificity	***p*** value
Age (years)	65.5	0.721(0.644–0.799)	47.6%	85.5%	< 0.001
PLT (10^9^/L)	217.1	0.605(0.518–0.693)	70.2%	50.0%	0.021
ALT (U/L)	21.5	0.605(0.518–0.692)	57.1%	60.5%	0.022
AST (U/L)	18.5	0.594(0.505–0.682)	67.9%	52.6%	0.041
CRP (mg/L)	11.2	0.746(0.669–0.823)	85.7%	59.2%	< 0.001
D-dimer (mg/L)	1.0	0.605(0.586–0.755)	69.0%	67.1%	< 0.001

*ROC* receiver operating characteristic, *CI* confidence interval, *PLT* platelet, *ALT* alanine transaminase, *AST* aspartate transaminase, *CRP* C-reactive protein

### Univariate analysis for the categorical variables

Table 4 shows the univariate analysis of the categorical variables of interest. Among the twenty-eight predictive variables, a total of eight factors (age, injury mechanisms, PLT, ALT, AST, CRP, D-dimer, and ASA score) were correlated with preoperative DVTs. Therefore, these eight factors were subjected to the multiple logistic regression analysis.

**Table 4:** Univariate analysis of categorical variables with interest

Variables	Number (%) of patients without DVT (***n*** = 76)	Number (%) of patients with DVT (***n*** = 84)	***p*** value
Gender (males)	26(34.2)	33(39.3)	0.506
Age (> 65 years)	11(14.5)	40(47.6)	< 0.001*
BMI (kg/m^2^)			0.573
< 18.5	1(1.3)	1(1.2)	
18.5–23.9	26(34.2)	22(26.2)	
24.0–27.9	29(38.2)	41(48.8)	
≥ 28.0	20(26.3)	20(23.8)	
Living place (rural)	51(67.1)	53(63.1)	0.595
Diabetes mellitus	20(26.3)	20(23.8)	0.715
Hypertension	21(27.6)	25(29.8)	0.766
Cardiovascular diseases	14(18.4)	15(17.9)	0.926
Previous surgery in any site	29(38.2)	22(26.2)	0.105
Smoking	6 (7.9)	8(9.5)	0.716
Alcohol consumption	6(7.9)	11(13.1)	0.286
Injury mechanisms (high energy)	29(38.2)	50(59.5)	0.007*
Fracture side (left)	45(59.2)	48(57.1)	0.791
Concurrent fractures (≥ 2 sites)	14(18.4)	20(23.8)	0.405
Fracture classification			0.578
Type A	44(57.9)	49(58.3)	
Type B	11(14.5)	8(9.5)	
Type C	21(27.6)	27(32.1)	
ASA score			0.001*
I–II	59(77.6)	43(51.2)	
III–V	14(22.4)	41(48.8)	
HGB (< lower limit)	23(30.3)	37(40.0)	0.072
RBC (< lower limit)	44(57.9)	58(69.0)	0.143
WBC (> 10 × 10^9^/L)	31(40.8)	31(36.9)	0.614
PLT (> 217 × 10^9^/L)	38(50.0)	59(70.2)	0.009*
TP (< 60 g/L)	36(47.4)	44(52.4)	0.527
ALB (< 35 g/L)	34(44.7)	45(53.6)	0.264
ALT (> 21.5 U/L)	30(39.5)	48(57.1)	0.026*
AST (> 18.5 U/L)	36(47.7)	57(67.9)	0.009*
CRP (> 11 mg/L)	31(40.8)	72(85.7)	0.001*
PT (> 12.5 s)	10(13.2)	13(15.5)	0.676
APTT (<28 s)	25(32.9)	38(45.2)	0.111
FIB (> 4.4 g/L)	11(14.5)	18(21.4)	0.254
D-dimer (> 1.0 mg/L)	25(32.9)	59(70.2)	< 0.001*

*Statistical significance

*DVT* deep vein thrombosis, *BMI* body mass index, *ASA* the American Society of Anesthesiologists, *HGB* hemoglobin, reference range: female, 110–150 g/L, male, 120–160 g/L; *RBC* red blood cell, reference range: female, 3.5–5.0 × 10^12^/L, male, 4.0–5.5 × 10^12^/L, *WBC* white blood cell, *PLT* platelet, *TP* total protein, *ALB* albumin, *ALT* alanine transaminase, *AST* aspartate transaminase; *CRP* C-reactive protein, *PT* prothrombin time, *APTT* activated partial thromboplastin time, *FIB* fibrinogen

### Multiple logistic regression analysis

Table 5 shows the final variables of the multiple logistic regression analysis. It is shown that >65 years of age (odds ratio [OR] 4.390; 95% confidence interval [CI] 1.727–11.155; *p* = 0.002), CRP > 11 mg/L (OR 4.158; 95% CI 1.808–11.289; *p* = 0.001), PLT > 217 × 10^9^/L (OR 2.547; 95% CI 1.068–6.073; *p* = 0.035), D-dimer > 1.0 mg/L (OR 3.496; 95% CI 1.483–8.237; *p* = 0.004), and an ASA score of III-V (OR 2.753; 95% CI 1.216–6.729; *p* = 0.026) were the five independent risk factors for preoperative DVT. The Hosmer-Lemeshow test showed adequate fitness (*χ*^2^ = 12.837; *p* = 0.118).

**Table 5:** Multivariate analysis of factors associated with preoperative DVTs

Variables	Odds ratio	95% CI	***p*** value
Age > 65 years	4.390	1.727–11.155	0.002
ASA (III–V)	2.753	1.216–6.729	0.026
CRP > 11 mg/L	4.158	1.808–11.289	0.001
PLT > 217 × 10^9^/L	2.547	1.068–6.073	0.035
D-dimer > 1 mg/L	3.496	1.483–8.237	0.004

*DVT* deep vein thrombosis, *CI* confidence interval, *ASA* the American Society of Anesthesiologists, *CRP* C-reactive protein, *PLT* platelet

## Discussion

This study revealed that preoperative DVT after DFFs was 52.5% in in-hospital follow-ups, which is consistent with previous findings in studies on lower extremity trauma of the lower extremity [17, 18]. However, studies have documented a lower overall DVT rate in other fracture sites when compared to our findings [8–13]. This could be attributed to the fact that our study cohort involved aged patients (58.82 ± 16.01 years). Moreover, asymptomatic patients with calf muscular vein thrombosis were also involved. After adjusting for confounding variables, age, ASA score, CRP, PLT, and D-dimer were shown to be independent risk factors for preoperative DVT.

Old age is an established risk factor for thrombosis as extensively studied by several pieces of orthopedic literature [10, 19]. Age > 40 years is a significant DVT predictor following fractures below the knee [20]. A retrospective study performed by Shibuya and colleagues on 75,664 ankle fracture cases showed that the age of the study participants in the DVT group (51.9 ± 21.4 years) was statistically higher when compared to that of the participants in the non-DVTs group (43.7 ± 20.6 years) [21]. Furthermore, Zhang et al. in their study on preoperative DVT after hip fracture (OR = 1.03, 71.51 ± 14.53 years) found that age was an independent risk factor [9]. In our study, the optimal cut-off value was found to be 65 years of age. Therefore, age > 65 years is an independent risk factor for preoperative DVT after closed DDF (OR = 4.39).

The optimum cut-off value for D-dimer was 1.0 mg/L, which was about twice that of the standard upper limit value (0.5 mg/L). A D-dimer value > 1.0 mg/L was correlated to a 3.50 times increased risk of preoperative DVT. Isolated D-dimer value tends to exhibit higher sensitivity, but often, the specificity is low in DVTs prediction. Moreover, D-dimer level is sensitive to age, infection, and cancer [22]. The combination of age with D-dimer as a critical value explicitly improved the predictive accuracy for DVT formation [23] and should be recommended. Compared to D-dimer, PLT volume is a common laboratory marker that is strongly correlated with coagulation. We established that patients with PLT > 217 × 10^9^/L had a 2.55-fold risk of developing preoperative DVT. Due to bone fracture and blood loss, elevated amounts of PLTs were released from the bone marrow, secreting biologically active substances such as thromboxane A2 and thrombomodulin [24, 25].

CRP is a protein biomarker for inflammation. It accompanies the inflammatory process. In 2003, the American Heart Association suggested that CRP levels be used in screening healthy adults for increased risk of coronary heart disease (levels above 3 mg/L) and for the detection of unsuspected and severe non-vascular diseases (levels above 10 mg/L) [26]. We found that CRP > 11 mg/L enhanced the probability of preoperative DVT by 4.16 times (95% CI 1.808–11.289). Studies have documented that CRP levels are elevated in acute DVT [27, 28]. Elevated levels of highly sensitivity CRP were closely correlated with the occurrence of DVT in ankle fracture patients before and shortly after the operation [28]. Furthermore, CRP has been proven to have a positive association with D-dimer, which could be attributed to the ability of the D-dimer and other fibrin degradation products to upregulate interleukin-6 synthesis, which promotes CRP synthesis [29].

The ASA classification system is a routinely applied evaluation scale for in-patients physical status, anesthetic, and surgical tolerance at the time of admission. Studies have documented the indispensable effects of the ASA score in the risk prediction of mortality and hip fracture complications [30]. In this study, the ASA score of III-IV correlated with quite a high risk of preoperative DVT (OR = 2.753). This finding was in concordance with that of a nationwide study that revealed an ASA score of > 2 (OR = 1.770) as being a significant risk factor for DVT after colorectal surgery [31]. A few published reports have documented an apparent correlation between ASA score and DVT formation. This could be attributed to the fact that ASA scores were obtained from evaluating the comprehensive medical histories and the judgment of the medical staff, which is not, however, an objective biomarker.

This study has three key highlights: (i) it used the largest prospective cohort of closed DFFs patients diagnosed by DUS for DVTs; (ii) DVT location analysis and their prognosis over 2 months were performed; and (iii) ROC analysis was performed to identify a highly sensitive cut-off value for continuous variables, and CRP > 11 mg/L was found to be an uncommon independent protective factor for preoperative DVT after closed DFFs. However, this study had some limitations. One, it was a single-center study that might not represent prevalent populations and, two, some variables, such as hidden blood loss or the number of days between the fracture and the operation that potentially determine the development of preoperative DVTs were not included.

## Conclusions

Elevated CRP, PLT, D-dimer, and ASA levels as well as age > 65 years were correlated with the increased risk of preoperative DVTs in adult patients with closed DFFs. Therefore, the prediction of preoperative DVTs can significantly be improved by identifying older patients over the age of 65 and, precisely establishing the biochemical cut-off values for CRP, PLT, ASA, and D-dimer.

## Data Availability

All the data will be available upon motivated request to the corresponding author of the present paper.

## Abbreviations

DVT: Deep vein thrombosis
BMI: Body mass index
HGB: Hemoglobin
RBC: Red blood cell
WBC: White blood cell
PLT: Platelet
TP: Total protein
ALB: Albumin
ALT: Alanine transaminase
AST: Aspartate transaminase
CRP: C-reactive protein
PT: Prothrombin time
APTT: Activated partial thromboplastin time
FIB: Fibrinogen
ROC: Receiver operating characteristic
CI: Confidence interval
ASA: The American Society of Anesthesiologists

## References

[1] Zhang Y (2016). Clinical epidemiology of orthopedic trauma.

[2] Philip M (2018). Patient mortality in geriatric distal femur fractures. J Orthop Trauma..

[3] Ng AC (2012). Trends in subtrochanteric, diaphyseal, and distal femur fractures, 1984-2007. Osteoporos Int..

[4] Nieves JW (2010). Fragility fractures of the hip and femur: incidence and patient characteristics. Osteoporos Int..

[5] Boyd AD, Wilber JH (1992). Patterns and complications of femur fractures below the hip in patients over 65 years of age. J Orthop Trauma..

[6] Christodoulou A (2005). Supracondylar femoral fractures in elderly patients treated with the dynamic condylar screw and the retrograde intramedullary nail: a comparative study of the two methods. Arch Orthop Trauma Surg..

[7] Godat LN (2015). Can we ever stop worrying about venous thromboembolism after trauma ?. J Trauma Acute Care Surg..

[8] Brill JB (2017). The rate of deep vein thrombosis doubles in trauma patients with hypercoagulable thromboelastography. J Trauma Acute Care Surg..

[9] Zhang BF (2018). Deep vein thrombosis in bilateral lower extremities after hip fracture: a retrospective study of 463 patients. Clin Interv Aging..

[10] Wang H (2018). Perioperative incidence and locations of deep vein thrombosis following specific isolated lower extremity fractures. Injury..

[11] Sloan M, Sheth N, Lee GC. Is Obesity Associated with Increased Risk of Deep Vein Thrombosis or Pulmonary Embolism After Hip and Knee Arthroplasty? A Large Database Study. Clin Orthop Relat Res. 2019;477:523–32. 10.1097/CORR.0000000000000615 PMC6382191 30624321

[12] Barker T (2016). Is there a link between the neutrophil-to-lymphocyte ratio and venous thromboembolic events after knee arthroplasty? A pilot study. J Orthop Traumatol..

[13] Meng H (2020). Incidence and risk factor for preoperative deep vein thrombosis (DVT) in isolated calcaneal fracture, a prospective cohort study. Foot Ankle Surg.

[14] Jupiter DC (2019). Acute Deep Venous Thrombosis and Pulmonary Embolism in Foot and Ankle Trauma in the National Trauma Data Bank: An Update and Reanalysis. J Foot Ankle Surg..

[15] Iskander GA (2006). Incidence and propagation of infrageniculate deep venous thrombosis in trauma patients. J Trauma..

[16] Rabinov K, Paulin S (1972). Roentgen diagnosis of venous thrombosis in the leg. Arch Surg..

[17] Decker S, Weaver MJ (2013). Deep venous thrombosis following different isolated lower extremity fractures: what is known about prevalences, locations, risk factors and prophylaxis?. Eur J Trauma Emerg Surg..

[18] Geerts WH (1994). A prospective study of venous thromboembolism after major trauma. N Engl J Med..

[19] Abelseth G (1996). Incidence of deep-vein thrombosis in patients with fractures of the lower extremity distal to the hip. J Orthop Trauma..

[20] Goel DP (2009). Prophylaxis of deep-vein thrombosis in fractures below the knee: a prospective randomised controlled trial. J Bone Joint Surg Br..

[21] Shibuya N (2012). Incidence of acute deep vein thrombosis and pulmonary embolism in foot and ankle trauma: analysis of the National Trauma Data Bank. J Foot Ankle Surg..

[22] Douma RA (2012). Using an age-dependent D-dimer cut-off value increases the number of older patients in whom deep vein thrombosis can be safely excluded. Haematologica..

[23] Schouten HJ (2013). Diagnostic accuracy of conventional or age adjusted D-dimer cut-off values in older patients with suspected venous thromboembolism: systematic review and meta-analysis. BMJ..

[24] Leader A, Pereg D, Lishner M (2012). Are platelet volume indices of clinical use? A multidisciplinary review. Ann Med..

[25] Han JS (2013). Increased mean platelet volume and mean platelet volume/platelet count ratio in Korean patients with deep vein thrombosis. Platelets..

[26] Gremmel T (2011). Soluble p-selectin, D-dimer, and high-sensitivity C-reactive protein after acute deep vein thrombosis of the lower limb. J Vasc Surg..

[27] Du YQ (2019). Correlation of interleukin-18 and high-sensitivity C-reactive protein with perioperative deep vein thrombosis in patients with ankle fracture. Ann Vasc Surg..

[28] Bakirci EM (2015). The role of the nonspecific inflammatory markers in determining the anatomic extent of venous thromboembolism. Clin Appl Thromb Hemost..

[29] Rumley A (2006). Effects of older age on fibrin D-dimer, C-reactive protein, and other hemostatic and inflammatory variables in men aged 60-79 years. J Thromb Haemost..

[30] Yin P, et al. Combination of red cell distribution width and American Society of Anesthesiologists score for hip fracture mortality prediction. Osteoporos Int. 2016;27:2077-7. 10.1007/s00198-015-3357-x 26975875

[31] Moghadamyeghaneh Z (2014). A nationwide analysis of postoperative deep vein thrombosis and pulmonary embolism in colon and rectal surgery. J Gastrointest Surg..

